# Addressing alpine plant phylogeography using integrative distributional, demographic and coalescent modeling

**DOI:** 10.1007/s00035-021-00263-w

**Published:** 2021-07-29

**Authors:** Dennis J. Larsson, Da Pan, Gerald M. Schneeweiss

**Affiliations:** 1 Department of Botany and Biodiversity Research, University of Vienna, Vienna, Austria; 2 Jiangsu Key Laboratory for Biodiversity and Biotechnology, College of Life Sciences, Nanjing Normal University, Nanjing, China

**Keywords:** Alpine plants, iDDC, Demographic modeling, Distribution modeling, Phylogeography

## Abstract

Phylogeographic studies of alpine plants have evolved considerably in the last two decades from ad hoc interpretations of genetic data to statistical model-based approaches. In this review we outline the developments in alpine plant phylogeography focusing on the recent approach of integrative distributional, demographic and coalescent (iDDC) modeling. By integrating distributional data with spatially explicit demographic modeling and subsequent coalescent simulations, the history of alpine species can be inferred and long-standing hypotheses, such as species-specific responses to climate change or survival on nunataks during the last glacial maximum, can be efficiently tested as exemplified by available case studies. We also discuss future prospects and improvements of iDDC.

## Introduction

Phylogeography is the study of how historical processes have shaped the geographic distributions of genetic lineages within a species or among closely related species (Avise 2000). As such, it answers questions such as species response to past glaciations, gene flow (or lack thereof) between geographically disparate populations or the role of vicariance versus dispersal in shaping distributions. As a field phylogeography has grown considerably since its introduction by Avise (1987) both in application and in methods utilized (Stehlik et al. 2001; Ikeda et al. 2009; He et al. 2013; Theodoridis et al. 2017). For a general overview on phylogeography and its methodological development, the reader is referred to the excellent reviews by Avise (2009), Knowles (2004, 2009), and Hickerson et al. (2010).

The progress of alpine phylogeography has seen our understanding of where alpine plants survived during the last glacial period (LGP, 115–11.7 ka) develop from tentative suggestions using qualitative data, usually derived from various nuclear and plastid markers under neutral evolution (Stehlik et al. 2001; Schönswetter et al. 2002; Kropf et al. 2003; Puşcaş et al. 2008; Ronikier et al. 2012; Gizaw et al. 2013; Wang et al. 2014), to explicit testing of hypotheses using sophisticated statistical models applied to large numbers of single nucleotide polymorphisms (SNPs) obtained from next generation sequencing (NGS) data (Theodoridis et al. 2017; Westergaard et al. 2019; Ikeda et al. 2020). This progress is also owed to the growing urgency to improve our insights into the phylogeographic histories of alpine plants: As many alpine species are under threat from the ongoing climate change (Freeman et al. 2018), comprehending how alpine plants survived past climate change may help us foresee if and how they will cope with future climate change. For instance, reduced snow depth and earlier timing of snowmelt induce changes in species-specific fitness of alpine plants growing in snowbeds, which may lead to changes in the species composition in those plant communities (Wipf et al. 2009). Other expected impacts due to a warming climate include changes in climatically suitable ranges (Hülber et al. 2020) and transformations of plant and pollinator compositions (Inouye 2020).

The phylogeography of alpine plants, however, is also interesting due to its model character. The rugged topography of mountains lead to complex interactions between temperature, precipitation, solar radiation, soil types, humidity, air pressure and the topography itself and gives rise to extreme environmental conditions that change dramatically over short distances, creating many different microhabitats (see Körner 2021 for an excellent overview on alpine plant ecology). Because alpine species are often restricted to their microhabitats, their distributions tend to be fragmentary and prone to considerable range shifts during changes in climate (Vargas 2003; Schönswetter et al. 2005; Schneeweiss and Schönswetter 2010). Range expansions and contractions and the associated changes in connectivity among populations result in complex phylogeographic patterns (Kadereit et al. 2004; Mráz et al. 2007; Dixon et al. 2009; Schneeweiss et al. 2017). By advancing our understanding of such patterns we can identify the general principles of how species distribute themselves over space and time.

In this review, we will outline the development of methodologies in alpine phylogeography, focusing on the relatively recent approach of integrative distributional, demographic and coalescent (iDDC) modeling. By integrating distributional data with spatially explicit demographic simulations and advanced statistical methods for model selection it becomes possible to test spatially explicit phylogeographic histories also in alpine plants, as already demonstrated by the few available studies. Finally, we will outline future prospects of the method and how it may be further improved.

## Available methods

### Interpretative phylogeography

Early phylogeographic studies on alpine plants used mostly qualitative methods, including ordinations such as principal coordinate analysis (PCoA; Schönswetter et al. 2002; Mráz et al. 2007), phylogenetic trees (Schönswetter et al. 2003; Puşcaş et al. 2008; Yang et al. 2012), hierarchical clustering using *STRUCTURE* (Pritchard et al. 2000) or related programs (Segarra-Moragues et al. 2007; Slovák et al. 2012), Mantel tests (Stehlik et al. 2001; Schönswetter et al. 2003), analysis of molecular variance (AMOVA; Stehlik et al. 2001; Kropf et al. 2003; DeChaine and Martin 2005; Ikeda and Setoguchi 2007; Chen et al. 2008) or the distribution of rare (i.e., being present in small proportion of individuals only) and private alleles (i.e., being restricted to usually a single population) of amplified fragment length polymorphisms (AFLPs) or of nuclear or plastid sequences (Mráz et al. 2007; Ronikier et al. 2012; Slovák et al. 2012; Gizaw et al. 2013; Wang et al. 2014). These observed genetic patterns were then compared to those expected under specific hypotheses. For instance, the distribution of different gene pools in geographically distinct regions may be taken as evidence for the location of Pleistocene refugia (Schönswetter et al. 2002; Massatti and Knowles 2014; Schönswetter and Schneeweiss 2019; Westergaard et al. 2019). However, this approach relies upon ad hoc interpretations (hence, the term “interpretative phylogeography”) and lacks objective means to assess whether a preferred hypothesis actually does fit better than alternative hypotheses. Here, we also include nested clade phylogeographic analysis (NCPA) in interpretative phylogeography. NCPA attempts to find an association between haplotypes and geographic location (Templeton et al. 1995), but even if an association is found, no explanation of what created the association (e.g. historical process) is provided (Knowles and Maddison 2002). Irrespective of that, NCPA was only rarely used in alpine phylogeography (Stehlik 2002; Bettin et al. 2007) and the method has become obsolete because of prohibitively high levels of false positives (Panchal and Beaumont 2010).

### Statistical phylogeography

#### From non-spatial to spatially explicit

To ascertain that a specific phylogeographic history explains observed genetic patterns it is necessary to show that the patterns can be replicated in a model that simulates the demographic conditions (population size changes, migration, etc.) of the specified phylogeographic scenario, but at the same time cannot be replicated in models of plausible alternative scenarios. The most popular way to do this is by simulating genealogies under the coalescent theory (Kingman 1982). This mathematical model describes how sampled alleles may have originated from the most recent common ancestor (MRCA) by merging (“coalescing”) them randomly backwards in time (Rosenberg and Nordborg 2002; for a detailed overview on coalescent theory see Rosenberg and Nordborg 2002 and Fu and Li 1999). Plausible genealogies can thus be simulated under models with conditions as expected under hypothesized phylogeographic histories and then be compared to observed genealogies within a statistical framework. Statistical frameworks are usually based on maximum likelihood or Bayesian Inference; common methods to evaluate models within these frameworks are Akaike information criterion (AIC; Akaike 1974) and Bayes factors (Jeffreys 1961), respectively (for more in depth information on the statistical frameworks see Marjoram and Tavaré 2006). Popular programs that apply these statistical frameworks include *FASTSIMCOAL2* (Excoffier et al. 2013), *DIYABC* (Cornuet et al. 2014) or *IMA3* (Hey et al. 2018). By testing models within a statistical framework it is possible to objectively determine the best supported model, which is the one considered most likely to have occurred. This approach was coined by Knowles and Maddison (2002) as “statistical phylogeography” and will be referred to as such henceforth. Examples of its application in alpine phylogeography include Theodoridis et al. (2017), Fu et al. (2018), Wang et al. (2019) and Ikeda et al. (2020). For further reviews on statistical phylogeography and how it is applied see Knowles (2004) and Knowles (2009).

For over a decade, it has been repeatedly argued that integrating spatial data in statistical phylogeography could greatly improve phylogeographic inferences (Knowles 2009; Hickerson et al. 2010; Chan et al. 2011; Alvarado-Serrano and Knowles 2014). One of the most popular types of spatially explicit data to be used are ecological niche models (ENMs), nearly exclusively based on climate data; for an overview on how ENMs can be integrated see Richards et al. (2007) and Alvarado-Serrano and Knowles (2014). Because ENMs, both for the present and the past, can provide a priori information on present and historical distributions, they can be used to inform the design of demographic models (e.g., number and relationships of demes), including the choice of priors for parameters (e.g., population size, divergence time), without making ad hoc assumptions (Carstens and Richards 2007; Knowles et al. 2007; Dépraz et al. 2008; Forester et al. 2013; Theodoridis et al. 2017). As this approach is not directly integrating the spatial information into the models, it is here referred to as “spatially-informed”.

As many phylogeographic patterns are driven by complex interactions that vary across the landscape, such as interactions with other species (Ortego and Knowles 2020), distributional shifts due to climate change (Knowles and Massatti 2017) or anthropogenic activity (González-Serna et al. 2019), simulating phylogeographic histories explicitly on a two dimensional landscape adds an additional layer of realism. Hereinafter, we will refer to those models as “spatially explicit”. Such spatially explicit simulations are often run on a “flat” landscape (Espíndola et al. 2012; Dellicour et al. 2014a, 2017); whereas the extent of the landscape is informed by environmental data such as ENMs, local differences in carrying capacity or factors affecting movement are not considered. There are two main types of spatially explicit demographic simulators: forward-time simulators and reverse-time simulators (Hoban et al. 2012). Specifically, forward-time simulators such as *SIMADAPT* (Rebaudo et al. 2013) or *NEMO* (Guillaume and Rougemont 2006) simulate the life history of individuals within populations, including birth, selection, mating system, mutation, migration and death, as time progresses forward. The high level of detail in these types of simulators enables accurate predictions of impact of ecological changes (e.g. climate change and human impact) on present populations, making them important tools in fields such as conservation biology and ecology (Yang et al. 2007; Bruford et al. 2010). The downside of forward-time simulators is that simulation on an individual level is relatively slow, especially when simulating over long time periods and with large population sizes. Furthermore, simulating from the past to the present requires hard to obtain knowledge about the past genetic composition of the initial individuals. In contrast, reverse-time simulators such as *IBMSIM* (Leblois et al. 2009) or *PHYLOGEOSIM 1.0* (Dellicour et al. 2014b) simulate from present time backwards using the previously mentioned coalescent theory, thus only requiring knowledge of present initial conditions. Moreover, because they simulate genealogies rather than individuals, they can simulate large population sizes over long periods of time faster than forward-time simulators. The downside of reverse-time simulators is that they usually cannot simulate complex life histories, making them less suitable for simulations where significant deviations from the Wright–Fisher model are possible (for an example, see Thalmann et al 2007). However, considering the speed of simulating over long time periods and the simplicity of only requiring knowledge of present initial conditions, reverse-time simulators appear to be more suitable for phylogeographic inferences. Most reverse-time simulators are only able to integrate spatial data to the extent of defining which cells are inhabitable. To our knowledge and at the time of writing, *SPLATCHE* (Currat et al. 2004) and it sequels, *SPLATCHE 2* (Ray et al. 2010) and *SPLATCHE 3* (Currat et al. 2019), are the only spatially explicit coalescent simulators that are able to integrate spatial data into models as a heterogeneous landscape.

#### The iDDC approach

Although spatially explicit demographic simulations have been applied to phylogeographic questions for some time (Currat and Excoffier 2004, 2005; Ray et al. 2005; François et al. 2008), a full integration of distributional data into these demographic models was first introduced by Knowles and Alvarado-Serrano (2010) and Brown and Knowles (2012). Their approach was later termed the integrative Distributional, Demographic and Coalescent (iDDC) method (He et al. 2013), whose major steps will be shortly outlined in the following.

**Fig. 1:**
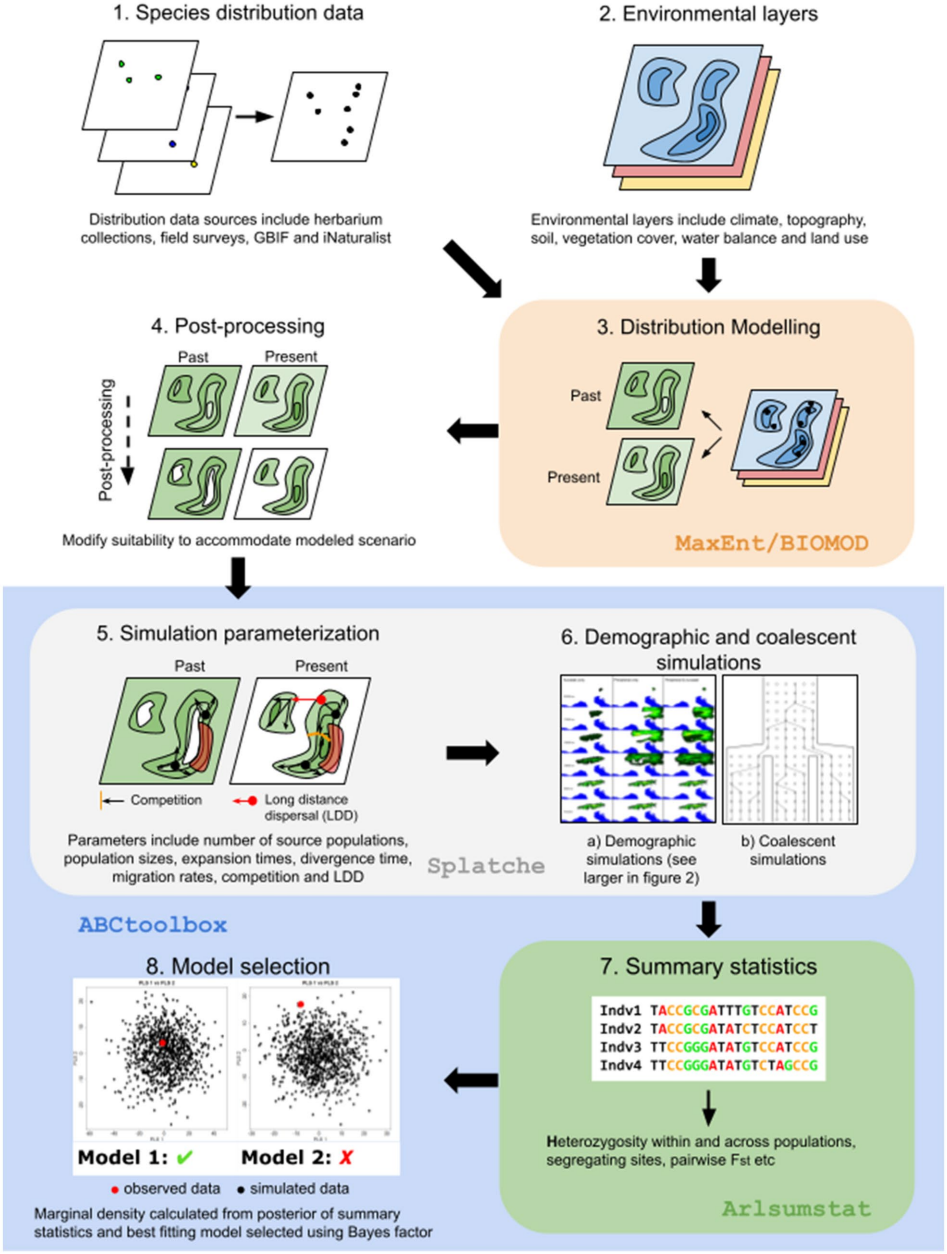
The iDDC workflow. Colored areas highlight central steps in the iDDC workflow and what programs are frequently used in that step. Below several steps are short descriptions of resources, parameters, etc. that can be used or modified. (1) Species distribution data and (2) relevant environmental data are collected and used to (3) conduct ecological niche modeling using programs such as *MAXENT* or *BIOMOD*; (4) the resulting models are modified to accommodate hypothesized scenarios (see Fig. 2). After defining friction layers (these are optional) and (5) setting up simulation parameters in *SPLATCHE*, (6) the demographic and coalescent simulations are run multiple times for each model and (7) summary statistics are estimated for each of these simulations; finally, (8) the best fitting model is selected

**Fig. 2:**
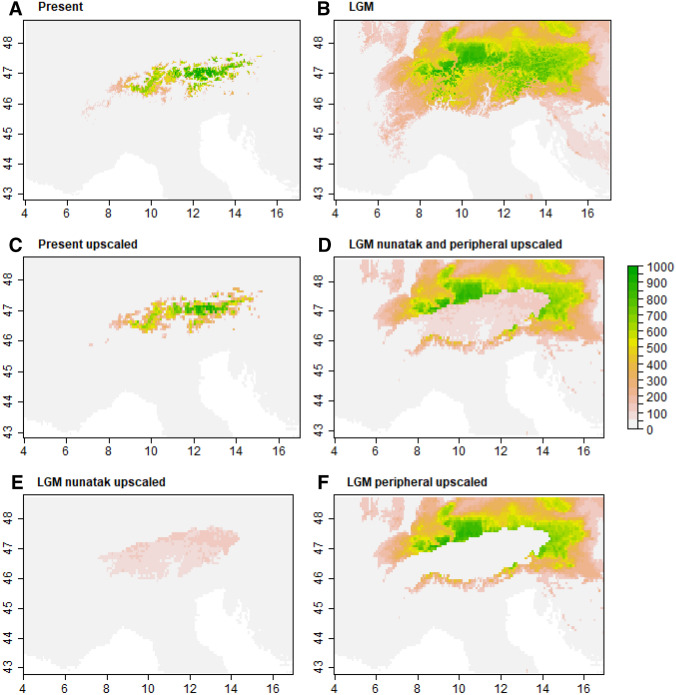
Raw and modified ecological niche models (EMNs) (data taken from Pan et al 2020). Unmodified ENMs for **A** the present and **B** the Last Glacial Maximum (LGM), respectively, are generated in *BIOMOD*. These are then modified by upscaling (reducing the resolution) and by setting habitat suitabilities (ranging from 0 to 1000) below a certain threshold (10% of maximum habitat suitability) to zero. In contrast to **C** the thus modified ENM for the present, the ENMs for the LGM were further modified: **D** all grid cells are available (i.e., both peripheral and interior refugia are permitted) with habitat suitabilities of grid cells inside the Alpine ice sheet reduced to 15% of the original habitat suitability; **E** only grid cells inside the Alpine ice sheet are available (i.e., only interior refugia are permitted) with the same reduction in habitat suitability as described before; **F** only grid cells outside the Alpine ice sheet are available (i.e., only peripheral refugia are permitted)

In the first step (corresponding to the “Distributional” part of iDDC), distribution data (step 1 in Fig. 1) and environmental layers (step 2 in Fig. 1) are used to construct ENMs (step 3 in Figs. 1 and  2A, B) for the relevant time periods (e.g., the last glacial maximum (LGM, 22 ka) and the present) using ecological niche modeling tools, such as *MAXENT* (Phillips et al. 2006) or *BIOMOD* (Thuiller et al. 2009; concerning best practices see Warren and Seifert 2011 and Elith et al. 2011 for *MAXENT* and Hao et al. 2019 for *BIOMOD*). The construction of ENMs assumes no changes in ecological niche since the past time period. This is a safe assumption when operating within tens of thousands of years, but may no longer hold when modeling longer time periods (Peterson 2011). The environmental layers usually include bioclimatic data from sources such as WorldClim (Hijmans et al. 2005; Fick and Hijmans 2017), but other factors that might restrict the range of the species, such as topography (Bemmels et al. 2016), can be included. Potential challenges for constructing accurate ENMs include local adaptation counteracting niche conservation (Smith et al. 2019) and correlation among environmental variables (Braunisch et al. 2013). For more information on ENMs in phylogeographic studies the reader is referred to the excellent review of Alvarado-Serrano and Knowles (2014).

Once the ENMs for the different time periods have been produced, they can be modified to accommodate specific hypotheses (step 4 in Fig. 1). This is, for instance, done by making areas uninhabitable (setting the habitat suitability in that area to zero) as seen in Fig. 2C–F, thus acting as barriers to dispersal during that time period.

**Fig. 3:**
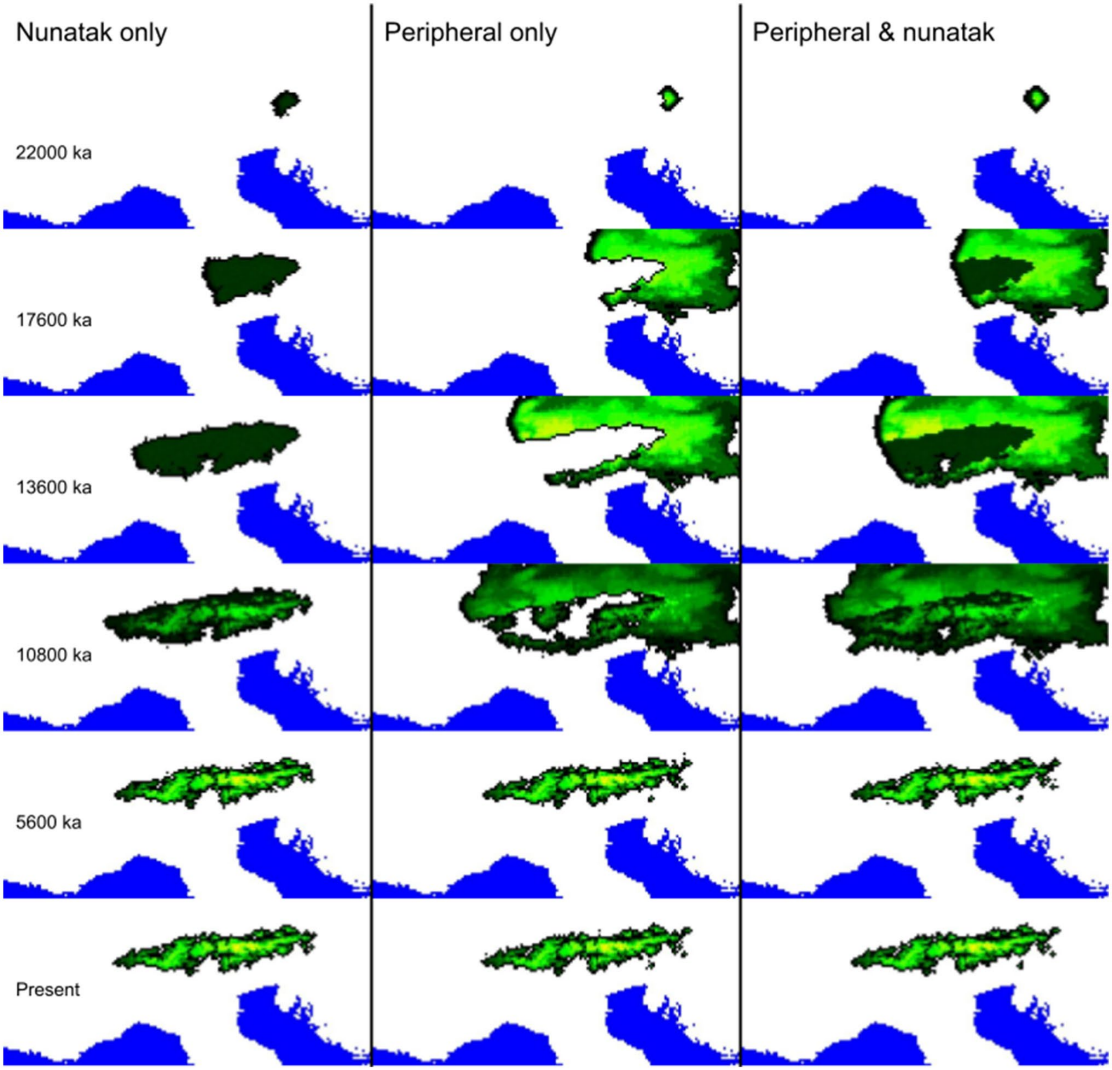
The extent of expansion (with eastern refugium as starting point) at selected time intervals during the spatially explicit demographic simulations in *SPLATCHE 3* for each of the three models tested by Pan et al. (2020)

The simulated space is made up of grid cells corresponding to or derived from the cells in the habitat suitability layers. Each of these grid cells is treated as a deme (a panmictic group of individuals). Habitat suitabilities derived from the ENM define the carrying capacities of inhabitable cells, i.e., the higher the habitat suitability the higher the carrying capacity; thus, the impact of environmental factors can be taken directly into account. Upon this landscape of cells the demographic simulations are run to simulate demographic expansions and contractions in two dimensions (corresponding to the “Demographic” part of iDDC). The habitat suitability layer can be changed at a defined time (step 5 in Fig. 1; Fig. 3), thus changing the carrying capacities of demes, to accommodate changes in climate or in other factors (e.g., barriers to migration). The tool (a script originally devised by He et al. 2013) used to convert ENMs from different time periods into the input files required by *SPLATCHE*, by translating the changes in habitat suitability between different time periods into classes of change, can only handle two time periods. The demographic simulation (step 6a in Fig. 1) is run forward in time, from a set time point in the past until present as seen in Fig. 3, to obtain a demographic history over the studied time period. In the third step (corresponding to the “Coalescent” part of iDDC), *SPLATCHE* uses this demographic history (population sizes at sampled demes, etc.) to run a coalescent simulation (step 6b in Fig. 1) backwards in time, resulting in a genealogy and thereupon simulated genetic data.

Summary statistics (step 7 in Fig. 1) over this genetic data are calculated using *ARLSUMSTAT* (Excoffier and Lischer 2010). The statistical framework used in iDDC is Approximate Bayesian Computation (ABC; Beaumont et al. 2002), which approximates the posterior distribution of parameters using rejection-sampling (e.g. only retain a specified number of best runs or only runs that are above a tolerance threshold) of summary statistics with regression adjustment and weighting (see Marjoram and Tavaré 2006, Csilléry et al. 2010 and Beaumont 2010 for overviews on ABC). ABC is thus well suited for cases where the evaluation of the likelihood function is too costly or analytically not possible for the full data, as is the case for complex problems, such as spatially explicit phylogeographic studies. For iDDC, ABC is done in *ABCTOOLBOX* (Wegmann et al. 2010), which is used jointly with *SPLATCHE* and *ARLSUMSTAT* (steps 5–8 in Fig. 1). Specifically, *ABCTOOLBOX* draws the parameter values for the demographic simulations in *SPLATCHE* (e.g., migration rates, ancestral population sizes) from prior distributions (instead of using fixed values as done in studies using ad hoc methods: Currat and Excoffier 2005; Ray et al. 2005) and sets the demographic simulation (and in consequence the genetic simulation) to be run multiple times (at least 10^5^ times, preferably longer) to achieve sufficiently good sampling of the parameters. As the number of summary statistics can be very high, possibly causing a “curse of dimensionality” effect (i.e., a high dimensional input causes large approximation errors: Bellman 1957), partial linear squares (PLS) components of the summary statistics are used instead (Wegmann et al. 2009). The type of genetic data available determines what summary statistics can be used, however there is no specific limitation on type of genetic data. For example, out of the seven iDDC studies cited in this review that use ABC, four used SNPs from restriction-site associated (RAD) sequencing (Massatti and Knowles 2016; González-Serna et al. 2019; Ortego and Knowles 2020; Pan et al. 2020), two used full sequence data from multiple nuclear loci (He et al. 2013; Knowles and Massatti 2017) and one used 13 genotyped nuclear microsatellite markers (Bemmels et al. 2016). The data and summary statistics used is highly dependent on the study. Summary statistics frequently used in the above studies include number of segregating sites for each population and across populations, mean heterozygosity across loci for each population and across populations, and pairwise population F_ST_.

Finally, the marginal density is calculated from the best fitting runs (commonly 0.5% of all simulations). Model selection (step 8 in Fig. 1) is done via Bayes factors calculated from the marginal densities of different models and is used to quantify how much better the best model is compared to the other models. Generally, a Bayes factor greater than 3.2 is considered significant, between 10 and 100 is considered strong and above 100 is decisive (Kass and Raftery 1995). In addition to the marginal density, the goodness-of-fit is assessed and expressed as a *p* value, which represents the proportion of the best fitting runs with a likelihood higher or equal to the observed data. A higher *p* value indicates a better fit between the simulated and the observed data. If the best model (highest marginal density) has a low *p* value, this model is just the best among a set of poor models, none of which can reliably explain the observed data; hence, the model set up has to be modified.

## iDDC case studies

The first time iDDC was applied to alpine plants was by Massatti and Knowles (2016). The authors tested whether the microhabitat preferences of *Carex nova* and *C. chalciolepis* (Cyperaceae), two sedges from the Southern Rocky Mountains, influenced their ability to survive and migrate through glaciated mountains during the LGM. Specifically, *C. nova*, which prefers wet microhabitats (moist subalpine and alpine swards, streams and lake margins; Ball and Reznicek 2021), was not expected to have been able to grow at higher altitudes due to the large accumulation of snow and ice inside drainages during the LGP. In contrast, *C. chalciolepis*, which prefers dry microhabitats (subalpine to alpine swards; Ball and Reznicek 2021), may have been able to inhabit ice-free ridges at higher altitudes, enabling this species to have been more interconnected during the glacial periods. To test these hypotheses the authors created two models, one where high altitude areas that were glaciated during the LGM were permeable (permeable model), albeit with low suitability, and one where these areas acted as barriers (barrier model). This was done by first reconstructing, for each species separately, ENMs for the LGM and for the present using *MAXENT* and then averaging the ENMs across the two species for each time period, resulting in one averaged ENM for the LGM and one averaged ENM for the present. Thus, differential outcomes of the models due to differences in the ENMs of the two species were avoided. Then the authors modified the ENM for those parts that were known to have been glaciated during the LGM to have habitat suitability reduced to 15% of the original suitability in the permeable model and to zero suitability in the barrier model. For each of the two models one million simulations were generated for each species. For *C. nova* the barrier model fitted significantly better to the observed data (Bayes factor: 22.69) than the permeable model. The reverse was true for *C. chalciolepis*, where the permeable model fitted better than the barrier model, yet only with weak support (Bayes factor: 2.84). The goodness-of-fit of the best models for both species was high (0.970 for *C. chalciolepis* and 0.844 for *C. nova*), indicating that the models performed well at reconstructing the observed data.

The second iDDC study on alpine plants was conducted on two species in the European Alps by Pan et al. (2020). The purpose of the study was to test the hypothesis of nunatak survival (i.e. survival on mountain peaks protruding above the continuous ice sheets), which has been suggested in multiple studies (Stehlik et al. 2002; Escobar García et al. 2012; Schönswetter and Schneeweiss 2019), but has rarely been tested explicitly (Westergaard et al. 2019). The two study species, *Pedicularis asplenifolia* (Orobanchaceae) and *Carex fuliginosa* (Cyperaceae), both grow in alpine swards and, especially the former, also in subnival communities. The authors hypothesized that these cold-adapted species could have survived the last LGM within the Alpine ice sheet on nunataks (alone or in addition to refugia at the periphery of the Alps). For each species, ENMs were created for the LGM and for the present using the R package *BIOMOD* (Thuiller et al. 2009, 2020). The ENMs from the LGM were then modified to accommodate three models: survival in the periphery of the Alpine ice sheet only (peripheral survival = Peri), survival both in the periphery of and on nunataks within the ice sheet (peripheral and nunatak survival = Peri + Nun), and survival only on nunataks within the ice sheet (nunatak survival = Nun). For Peri the suitability in ice-covered regions was set to zero, for Peri + Nun and Nun the suitability of ice-covered regions was set to 15% of the original, and for the Nun model additionally the suitability outside the ice-covered regions was set to zero. Due to computational limitations, Pan et al. (2020) only used one starting point (one refugium) in the demographic simulations. Thus, effectively six models were simulated for each species, where the Peri and Peri + Nun models were simulated two and three times, respectively, each time with a different location for the ancestral refugium (southern periphery, eastern periphery and central nunatak). Similar to Massatti and Knowles (2016), for each model one million simulations were generated. For *P. asplenifolia* the Peri_East_ + Nun model (eastern periphery plus central nunataks as refugia) fitted the observed data best, overwhelmingly better compared to the alternative models (Bayes factor > 100), and had a decent goodness-of-fit (*p* value of 0.736). However, for *C. fuliginosa* the results were less decisive. The Peri_East_ model (eastern periphery as refugium) was decisively better (Bayes factor > 100) than all other models except the Peri_East_ + Nun model, where the Bayes factor was only 5.58. For both models, the p-values were very high (0.993 and 0.992 for the Peri_East_ and the Peri_East_ + Nun model, respectively), indicating a good reconstruction.

The results of the two studies show that the iDDC method can be successfully used to contrast biologically informed hypotheses and provide insight into the phylogeographic history of alpine plants. Both studies suggest that alpine plants did not experience a universal response to Pleistocene climate change, but rather show species- or possibly “cohort”—(a group of species with similar ecological traits) specific responses, which are determined by adaptations to specific ecological conditions. The signal for this is particularly strong in the two North American sedges (Massatti and Knowles 2016), whose primary ecological difference concerns their microhabitat preferences. The good fit of the simulated data from the permeable model to the observed data in the dry adapted species and, likewise, for the barrier model in the wet adapted species is in line with the hypothesis that dry adapted species could have survived (also) on dry ice-free ridges at higher altitudes, whereas wet adapted species had to survive outside the ice sheet at lower altitudes. Although Pan et al. (2020) primarily investigated nunatak survival, the unambiguous support for nunatak survival in the species more tolerant to harsher conditions (as it can grow also in the subnival belt) and the unclear model choice for the species not tolerant to subnival conditions are indicative of differing response to climate change for the two species. However, as Pan et al. (2020) point out, the equivocal result in *C. fuliginosa* may also be the result of postglacial genetic swamping and the resulting genetic homogenization, which appears likely in the wind-pollinated sedge species. Either way, the study provides strong support in favor of the nunatak survival hypothesis, especially for species that grow in the subnival belt. Although it is undoubtedly true that the best model is not necessarily the correct one, the results of the two studies clearly point to the direction that there are species-specific responses to (Pleistocene) climate change.

## Outlook

The iDDC method has proven itself useful in progressing our understanding of the phylogeography of alpine plants, but there are a number of drawbacks where improvements are desirable. One of those issues concerns the rather coarse resolution used for the demographic simulations. As the number of grid cells in the demographic simulation directly impacts the run-time, it is necessary to upscale (i.e., decrease resolution) the geographic layers to reduce the number of cells, leading to a coarser resolution. This is expected to be a particular problem in the alpine and subnival zones, where habitat conditions often differ strongly even over short distances (Körner 2021), and such differences will be more easily lost with decreasing resolution. Randin et al. (2009) found that species persistence (survival under future climate conditions) in alpine plants was predicted to be higher in a high spatial resolution model (25 × 25 m) versus a low resolution model (16 × 16 km). The authors suggest that this is likely due to the higher resolution model better representing the rugged topography of mountains, which allows microrefugia that are overlooked in the low resolution model to be captured. Similarly, Trivedi et al. (2008) showed that habitat loss in montane plants is underestimated in a low resolution model compared to a high resolution model. A cross-scale comparison revealed that the low resolution model overestimated the thermal tolerance of mountain plants because cold high altitude areas were averaged out in the coarser model.

While higher resolution layers (30 arc seconds) do exist for the present (Karger et al. 2017) and the LGM (Karger et al. 2021) from CHELSA, their usefulness may be limited for several reasons. Firstly, to our knowledge only González-Serna et al. (2019) have been able to achieve a computationally tractable resolution for the ENM smaller than 5 arc minutes (González-Serna et al. 2019 used 2.5 arc minutes) in a study applying iDDC. Secondly, although the evaluation of LGM and mid-Holocene (6 ka) climate models against paleodata (ice-core, marine and terrestrial archives) shows that paleoclimate models are able to reproduce large-scale changes, these climate models tend to underestimate regional variability (Braconnot et al. 2012). For this reason, estimates of climate at a local and regional level are likely to be inaccurate. This is further corroborated in the benchmark of paleoclimate models by Harrison et al. (2014), who found that models tend to agree on the direction of change, but differ in the amplitude of change. Thirdly, the microhabitats that many alpine plants are dependent on are usually shaped by factors that vary on a very small scale (meter-by-meter, Körner 2021), a level of resolution impossible to be modeled for past climates. Downscaling (i.e., increasing resolution) of paleoclimate models is a notoriously complex process (Lima-Ribeiro et al. 2015; Beyer et al. 2020; review by Harris et al. 2014), where minor biases on a global scale can create major biases on a regional scale (see the jet stream example of Hall 2014). While Hall (2014) mention a number of cases where downscaling can result in more accurate estimates, there is no guarantee that models on a scale relevant for alpine plant microhabitats will be accurate. Although the difficulties in obtaining paleoclimate data at a resolution relevant for alpine species may intuitively appear detrimental, the situation is not that bleak. For many alpine plants the most important phylogeographic patterns tend to occur over relatively large geographic distances, for example across mountain ranges (Schönswetter et al. 2005; Alvarez et al. 2009). Capturing microhabitats at a local scale certainly is needed to accurately estimate local population sizes during past climates, but for most species, except the geographically most strongly restricted ones, accurately recreating the overall habitat connectivity will be far more important. Therefore, for alpine plants distributed across mountain ranges and mountain systems the currently available relatively coarse resolution is sufficiently informative.

Connected to the issue of spatial resolution is temporal resolution (but note the current restrictions concerning the number of different time periods described in section *The iDDC approach*). Being able to cover time periods other than the LGM would likely improve the simulation of the phylogeographic history of alpine species, especially since time periods such as “the last glaciation” were climatically rather heterogeneous (Andersen et al. 2004; Jouzel et al. 2007). Both Paleoclim (Brown et al. 2018) and Worldclim offer bioclimatic variables for the last interglacial (LIG, (130–115 ka), and data from Paleoclim extend as far back as the Pliocene (ca. 3.3 Ma). Whereas Worldclim does have bioclimatic variables for the mid-Holocene, Paleoclim and CHELSA cover more time points between the LGM and the present; CHELSA even has model data for every 100 years from 21 to 1 ka. Beyer et al. (2020) similarly offer bioclimatic variables for more time periods, however with greater focus on the LIG, the LGP and the Holocene, by providing bioclimatic variables for every 2000 years between the LIG (120 ka) and the LGM and every 1000 years between the LGM and present. However, this dataset is only available at 30 arc minutes resolution and would certainly need to be downscaled for use with alpine plants (i.e., in topographically complex regions like mountains). Even if paleoclimate data would be available in optimal temporal resolution going far beyond the LGM, it has to be kept in mind that when modeling ecological niches hundreds of thousands of years into the past the assumption of niche constancy is more likely to be violated.

Another issue concerns the type of data used for ecological niche modeling. In both Pan et al. (2020) and Massatti and Knowles (2016) only climatic data was used, but other types of environmental data are expected to be relevant as well and can be implemented. For example, snowpack was included as an environmental variable in Forester et al. (2013) for the alpine plant *Rhodiola integrifolia* (Crassulaceae) and subsoil pH was included in de Melo et al. (2016) for the dry tropical forest tree *Tabebuia rosealba* (Bignoniaceae). Piedallu et al. (2013) showed that using soil water balance (calculated using soil and precipitation data) instead of precipitation or climate related proxies for water balance significantly improved the distribution model of the majority of 37 tree species, although somewhat unevenly; specifically, 71–100% of species, ranging from hygrophilous to xerophilous ones, had improved distribution models (the strongest in hygrophilous species), whereas for mesophilous species only 25% of species had improved distribution models. Data for environmental parameters beyond climate data are available, for example soil data on a global scale in the Harmonized World Soil Database v 1.2 (Fischer et al. 2008) or on a European scale in the European Soil Database v 2 (ESDB 2004; Panagos 2006). Their relevance for alpine habitats may, however, be limited due to insufficient spatial resolution. Despite the intuitive relevance of including more environmental data, improving ecological niche models beyond bioclimatic variables may not improve iDDC models. In an iDDC study by Bemmels et al. (2016) on canyon live oak (*Quercus chrysolepis*, Fagaceae), two simple models including either only climate variables or drought/growth related variables had significantly higher marginal densities than all other models, including those that contained additional variables (e.g. trade offs between growth rate and cold tolerance, topography). Whereas this suggests that including more variables does not necessarily make the model better, more studies are necessary to assess to what extent the performance of iDDC models are affected by the quality of ENMs based on different environmental variables.

Although the most common approach to construct competing models in iDDC is to modify the ENMs (step 4 in Fig. 1), there are other functionalities in *SPLATCHE* that may be used for hypothesis testing. For example, a friction layer can be used to modify the rate of migration into demes, allowing testing of hypotheses that only concern rate of migration, but not habitat suitability. For example, in areas with directional winds, migrating upwind might be difficult for wind dispersed species, even though the habitats are suitable. In this case, the hypothesis could be tested by defining a friction layer to make demes upwind of the source population more difficult to migrate into. Friction layers can also be used to facilitate migrations, allowing spatially distributed factors to be tested that may act as dispersal corridors, such as roads and rivers (Johansson et al. 1996; Tikka et al. 2001). Another functionality available since *SPLATCHE* 2 that could be used for hypothesis testing is its two-populations mode, which can be used to test interactions, including competition, between lineages (e.g., species or subspecies). An example where this might be relevant is *Phyteuma globulariifolium* (Campanulaceae). In this species, the boundary between its two major lineages (essentially corresponding to previously identified subspecies) in the European Alps is largely defined by wide inhospitable valleys, except in the northern part where there is no clear ecological or geomorphological barrier (Schönswetter et al. 2002). By testing models with or without competition, a better understanding of what maintains the genetic barrier between the two subspecies could be gained. In contrast to previous versions of the program, *SPLATCHE* 3 can also simulate long distance dispersal (LDD), which could be used to test whether currently isolated populations of alpine plants are the result of LDD or of vicariance following upward range shifts. It remains to be seen, though, whether currently available data, both environmental and genetic, are of sufficient resolution to distinguish such scenarios.

A certain disadvantage of *SPLATCHE* is that functions for demographic and genetic simulations are hard-coded, potentially restricting the range of questions possible to address with iDDC. Using a set of modular libraries, such as are becoming available via *QUETZAL* developed by Becheler et al. (2019), may enable improved integration and model refinement, allowing more hypotheses with greater detail and accuracy to be tested.

## Conclusion

Despite some potential drawbacks, which are mostly technical (e.g., low resolution of environmental layers), the iDDC approach has opened up new avenues of phylogeographic research by the integration of spatially explicit demographic simulations with distributional data. This is well exemplified by the few available case studies, as both Massatti and Knowles (2016) and Pan et al. (2020) have furthered our understanding of the phylogeographic history of alpine species by testing both long-standing hypotheses in alpine phylogeography (nunatak versus peripheral survival) and new hypotheses on how past pre-adaptation to different ecologies may facilitate differential responses to recent climate change. We expect that with iDDC’s ability to address new phylogeographic questions using spatially explicit demographic modeling and the increasing ease of obtaining large-scale genetic data from alpine plants, approaches like the iDDC will play a central role in alpine phylogeography in the future.
